# Pre-Treatment Effects on Chemico-Physical Characteristics of Argan Press Cake Used for Bread Production

**DOI:** 10.3390/foods14081315

**Published:** 2025-04-10

**Authors:** Asma El Kaourat, Hasnae Choukri, Badr Eddine Kartah, Ahmed Snoussi, Giuseppe Zeppa, Aouatif Benali, Mouna Taghouti, Hanae El Monfalouti

**Affiliations:** 1 Laboratory of Plant Chemistry, Organic and Bioorganic Synthesis, Faculty of Sciences, Mohammed V University in Rabat, 4 Avenue Ibn Battouta, Rabat B.P. 1014 RP, Morocco; asma.elkaourat@gmail.com (A.E.K.); b.kartah@um5r.ac.ma (B.E.K.); 2 International Center for Agricultural Research in the Dry Areas (ICARDA), Rabat 10112, Morocco; 3 Innovation and Valorisation Laboratory for a Sustainable Food Industry, Higher School of Food Industries of Tunis ESIAT, University of Carthage, 58 Av. Alain Savary, Tunis El Khadra 1003, Tunisia; ahmed.snoussi@esiat.ucar.tn; 4 Department of Agriculture, Forestry and Food Sciences (DISAFA), University of Turin, 10095 Grugliasco, Italy; 5 National Institute of Agricultural Research (INRA), Rabat-Instituts, Rue Hafiane Cherkaoui, Rabat 10101, Morocco

**Keywords:** argan press cake, pre-treatment, nutritional enhancement, composite flour, intrinsic structure bread, waste management

## Abstract

Argan oil is known worldwide for its nutritional, therapeutic, and cosmetic benefits. However, the extraction process produces 40–50% of argan press cake (APC), which is rich in protein, fiber, and minerals. Despite its nutritional potential, the high saponin content of APC imparts a bitter taste and anti-nutritional properties, making it unsuitable for human consumption and often wasted. This study addresses this issue by using boiling treatments with citric acid (CA) and distilled water (DW) to reduce the saponin content while evaluating the impact on APC quality. In addition, this study explores, for the first time, the incorporation of treated argan press cake, APC-CA and APC-DW, at different levels (5%, 10%, 15%, and 20%) into whole wheat flour (WWF) for bread production to improve the nutritional profile. The results indicate that both treatments significantly reduce saponin content while maintaining nutritional quality comparable to untreated APC. This includes a 50% reduction in phytic acid levels. The absence of tryptophan fluorescence emission was observed in APC-CA, which may be related to chemical degradation or interactions with other molecules. The substitution of APC-CA and APC-DW increased the protein of composite flours in a level-dependent manner. At substitution levels up to 10%, APC-CA and APC-DW positively influenced the technological properties of the bread. This study demonstrates the potential of APC to improve the nutritional value of bread and supports zero-waste initiatives by reusing by-products.

## 1. Introduction

The argan tree (*Argania spinosa* (L.) Skeels), an endemic species of southwestern Morocco, is cultivated by 235 development units and involves 2 million beneficiaries in rural areas. There are 16 production areas, 375 cooperatives, 450 companies, six EIGs, and three unions [1]. It is a multipurpose tree that plays a crucial socio-economic and environmental role for the Moroccan Berber population. Argan oil, the main important product, is internationally known for its nutritional properties and its therapeutic and cosmetic benefits, which are highly valued and sought after by the cosmetic industry [2,3]. According to Zeghlouli et al. [4], 35 kg of argan fruit yields about 1 L of argan oil, so the production process generates substantial amounts of biomass, including about 39% pulp, 53% shells, and 6% seeds [5,6,7]. During the argan oil production process, 2% of argan press cake is generated [8], which is mostly wasted or used as animal feed [9].

In recent years, food by-product valorization for edible purposes has gained considerable attention due to its potential applications in developing functional foods and managing agro-industrial waste. Various food by-products contain significant amounts of secondary metabolites, protein, dietary fibers, and essential nutrients, making them suitable for improving the quality and health benefits of food products [10], such as grape pomace [11], olive mill waste [12], citrus peels [13], and rice bran [14], which have successfully contributed to enhanced nutritional value and functional properties in food formulations.

Several studies highlight the importance of argan press cake (APC). It has various applications, including shampoo production, treatment of sprains and scabies, and wound healing. In addition, it has an inhibitory effect on melanogenesis in B16 melanoma cells [15]. APC is also used as a nano-adsorbent [3], and there are ongoing efforts to valorize it by extracting the saponins. Various applications are being investigated, including emulsifying [2] and anti-hyperglycemic agents [4].

The APC contains high levels of protein (41–47%), dietary fiber (16–17%), carbohydrate (9–12%), and crude lipid (18–23%) [4,16]. However, its use in nutrition is limited due to the high saponins content (4.2%) [17], which have been shown to inhibit digestive enzymes, such as amylase, glucosidase, trypsin, chymotrypsin, and lipase. These inhibitory effects could potentially contribute to health problems associated with indigestion [18] and reduce the availability of essential micronutrients in the human diet [19,20].

For this purpose, this study aimed to reduce the saponin content of argan oil cake (APC) by using hot water or a hot citric acid solution. The efficacy of these solutions was evaluated by incorporating the treated APC into whole wheat flour to produce bread. This study gives an insight into the use of argan oil cake as an ingredient that can reduce waste from argan oil production

## 2. Materials and Methods

### 2.1. Chemical Reagents

All chemical reagents were of analytical grade and were provided by Sigma-Aldrich, St. Louis, MO, USA. Nitric acid (HNO_3_, 65%), hydrogen peroxide (H_2_O_2_, 30%), hydrochloric acid (HCl, 37%), sodium dodecyl sulfate (SDS, 99%), and lactic acid (98%) were used.

### 2.2. Raw Materials

The whole wheat flour (WWF) used in this study was produced from ‘LINA’, a bread wheat variety (*Triticum aestivum* L.) obtained from the INRA (National Institute for Agronomic Research) in Rabat (Morocco). The APC was obtained from an argan oil-producing cooperative located in the Taroudant region of southwestern Morocco. A cyclone mill (UDY Corporation, Fort Collins, CO, USA) was used to obtain fine flour with uniform size of 1 mm.

### 2.3. APC Treatment

The saponin content of APC was reduced using hot distilled water (DW) or a hot solution of citric acid (1%; CA). This treatment was carried out at 90 °C for 10 min using a reflux system. After the treatment, the solutions were separated from the APC by vacuum filtration. The APC was rinsed with distilled water and dried in a hot air oven at 40 °C. The resulting powder samples were stored in hermetically sealed plastic containers at 25 °C for future use.

### 2.4. Proximate Analysis

Proximate analysis of whole wheat flour and mixed flours was performed according to the approved methods of the American Association of Cereal Chemists (AACC, 2000) [21]. The Kjeldahl method, used to measure total nitrogen, consists of digesting 1 g of each powdered sample with sulfuric acid. The resulting extract was then alkalized with 40% sodium hydroxide. Distilled ammonia was captured in a 4% boric acid solution and then titrated with 0.1 N hydrochloric acid. The nitrogen content was then multiplied by the conversion factor to determine protein content (Method 46-13). Acid hydrolysis with 1.25% H_2_SO_4,_ followed by alkaline hydrolysis with 1.25% NaOH, was used to determine crude fiber (Method 32-10). Total ash was determined by high-temperature combustion at 585 °C until a constant weight was achieved. The residue is then cooled to room temperature and weighed accordingly. (Method 08-01). Moisture content was determined by drying the samples at 130 °C to constant weight (Method 44-15). Samples were exhaustively extracted by Soxhlet using hexane as solvent to determine crude fat (Method 30-25). All analyses were performed in triplicate.

### 2.5. Mineral Content

Mineral concentrations were measured according to a previously published method [22]. Briefly, 0.5 g of each sample was weighed into a glass tube, and then 6 mL of nitric acid (HNO_3,_) was added. The prepared mixture was then placed in the digestion block (QBlock series, Questron Technologies Corp, Mississauga, ON, Canada), and after 1 h of heating at 90 °C, 3 mL of hydrogen peroxide (H_2_O_2_) was added to each tube. The mixture was further heated at 90 °C for 15 min. Then, 3 mL of 6 M hydrochloric acid (HCl) was added to each sample tube. At the end, a clear solution was obtained, which was then transferred to a volumetric flask and diluted with ultrapure water to a volume of 10 mL. Calcium, zinc, and iron were analyzed using the Inductively Coupled Plasma ICP Ultima 2 (Horiba Jobin Yvon, Longjumeau, France), and calibration curves were constructed using serial dilutions from 0.1 to 100 mgL^−1^.

### 2.6. Phytic Acid Content

For the evaluation of phytic acid content in a 50 mL vial, 1 g of each sample was digested with 20 mL of 0.66 M HCl solution and stirred overnight at room temperature to release the inorganic phosphorus from the phytic acid. The phosphorus content was determined using a Megazyme Phytic Acid Assay Kit (Megazyme, Wicklow, Ireland). The phytic acid content of the samples was calculated using the following formula:

(1) $$Phytic\;acid=\frac{Phosphorus\left(g/100g\right)}{0.282}$$

The molar ratios phytic acid/iron and phytic acid/zinc were calculated according to the following equation:

(2) $$PA/MNratio=\frac{PA/MW_{AP}}{MN/MW_{MN}}$$

where *PA* is phytate acid content; *MW_PA_* is *PA* molecular weight (660.04 g mol^−1^); *MN* is micronutrient content (zinc or iron); and *MW_MN_* is micronutrient molecular weight (Zn = 65.4 g mol^−1^; Fe = 55.85 g mol^−1^).

### 2.7. Saponin Content

Twenty grams of each sample was placed in a conical flask with 100 mL aqueous ethanol (80/20 *v*/*v*) and then heated in a water bath (Büchi Labortechnik AG, Flawil, Switzerland) at approximately 55 °C for 4 h with continuous stirring. The mixture was filtered through a paper filter to separate the extract from the residue. The residue was re-extracted with 200 mL of aqueous ethanol (80/20 *v*/*v*). The two extracts were mixed and then reduced to 40 mL using a water bath at 90 °C. Forty milliliters of the extract was transferred to a 250 mL separating funnel, and then 20 mL of diethyl ether was added. The mixture was shaken vigorously to allow the separation of the saponins into the aqueous phase, and the ether layer was discarded. The purification process was repeated by shaking the mixture again with diethyl ether. Then, 60 mL of n-butanol was added to the aqueous layer obtained in the previous step. After shaking the mixture, the n-butanol extract was washed twice with 10 mL of 5% aqueous sodium chloride. The remaining solution obtained in the previous step was heated in a water bath to evaporate the solvent and to remove the n-butanol. Finally, the resulting sample was dried in an oven until a constant weight was obtained. The saponin content of the dried sample was then calculated [23].

### 2.8. Fluorescence Spectroscopy

Fluorescence analysis was performed using a Fluoro-Max-4 spectrofluorometer (Thermo-Scientific, Horiba, France), equipped with a 150 W xenon lamp and a Haake A25 AC 200 temperature controller, with the angle of incidence of the excitation radiation set at 60° to reduce the reflected light. Fluorescence emission spectra were recorded at 20 °C between 270 and 650 nm, with excitation wavelengths between 250 and 602 nm.

### 2.9. Infrared Spectroscopy Measurements

Fourier transform infrared (FTIR) spectroscopy was used to provide information on the treatment effect on the protein network of APC. Spectra were recorded at room temperature (25 °C) between 4000 and 400 cm^−1^ with a resolution of 4 cm^−1^ using an IRTracer-100 Fourier transform spectrometer (Shimadzu, Duisburg, Germany) mounted with an attenuated total reflection (ATR) accessory equipped with a handle (Pike Technologies, Inc., Madison, WI, USA). A diamond crystal was used in the ATR cell. Before each analysis, a reference spectrum was recorded, and the crystal was cleaned with ethanol and ultrapure water after each sample was analyzed. Further data processing was performed according to [24] to obtain the second derivative function of the spectral region between 1550 and 1700 cm^−1^. The relevant contribution of the obtained sub-bands was calculated by the curve fitting method using the free software MagicPlotStudent 2.9.3.

### 2.10. Breadmaking

The APCs were used to substitute whole wheat flour in the bread production at four concentrations (5, 10, 15, and 20%). For 300 g of whole wheat flour or mixed flours, 9.0 g of olive oil, 12 g of sucrose, 6 g of sodium chloride, and 95 mL of water were added. The breads were prepared using a household bread maker Backmeister 68511 (Unold, Hockenheim, Germany). Each sample received 3% freeze-dried yeast and underwent a “basic” program with the following steps: preheating for 17 min, initial kneading for 5 min, secondary kneading for 13 min, first proofing for 45 min, smoothing for1 min g, second proofing for 18 min, another 1 min of smoothing, third proofing for 45 min, and finally, baking for 55 min.

### 2.11. Technological Parameters

Measurement of flour and bread color was conducted using a Chroma Meter CR 400 (Konica Minolta, Carrières-sur-Seine, France), which was calibrated with a white calibration plate. According to the international standard ISO 5529, the SDS sedimentation test is used to measure gluten resistance. This method involves dispersing 1 g of each sample in a solution containing 6 mL of 3% sodium dodecyl sulfate (SDS, 99%) and 6 mL of 1.3 N lactic acid (98%) with bromophenol blue as an indicator. The test is based on measuring the volume of the sediment formed after stirring and swelling [25].

### 2.12. Mixograph

The mixing properties of wheat flour without and with the addition of APC flour were evaluated using a mixograph (National Mfg. Co., Lincoln, NE, USA). Water absorption, mixing time, and peak height of the tested composite flours were measured according to [26].

### 2.13. Loaf Volume

Loaf volume was determined using replacement rapeseed seeds according to the Method 10-05 (AACC, 2000) [21]. Each loaf of bread was placed in a 4 dm^3^ beaker, and rapeseed was added from a graduated cylinder until the beaker was full, and the volume of the bread was determined.

### 2.14. Image Analysis for Crumb Characteristics

Bread images were captured using CanoScan LiDE 220 scanner (Canon, Tokyo, Japan) and stored in JPEG format. Image analysis was performed using ImageJ software (version 1.46d). The center of each section was cut into a 10 × 10 cm^2^ square and subjected to binarization analysis. The software measured the total number of cells, total cell area, average cell area, and porosity, which is defined as the ratio of cell area to total area.

### 2.15. Statistical Analysis

The results are presented as the mean (n = 3) ± standard deviation. ANOVA and Tukey post hoc test were used to compare differences between means at *p* < 0.05 using the SPSS software version 25.0. 

## 3. Results and Discussion

### 3.1. APC Characteristics

#### 3.1.1. Nutritional Content

A significant difference (*p* < 0.05) was observed between untreated (UAPC) and treated APC samples in several parameters (Table 1). In particular, the saponin content showed a significant reduction, with decreases of approximately 83% and 75% for APC-CA and APC-DW, respectively, compared to the initial value in UAPC. This can be attributed to the effectiveness of heat treatment in reducing saponin content, as demonstrated by numerous studies aimed at reducing antinutritional composition [22,27]. The use of a citric acid solution was found to be more effective than distilled water in reducing saponin levels. This can be explained by the ability of saponins to form complexes with metal ions [28,29]. For example, citric acid acts as a chelating agent and forms complexes with metal ions [30]. Phytic acid, also known as phytate or myoinositol 1,2,3,4, S,6-hexakis dihydrogen phosphate, is a common component of legumes, seeds, and grains. Phytic acid can reduce the bioavailability of minerals and the digestibility of proteins and carbohydrates by inhibiting the normal activity of digestive enzymes such as pepsin, trypsin, and amylase. Phytic acid acts as a chelating agent for cations and a phosphorus storage agent in many seeds [31,32]. In our study, phytic acid levels were reduced by approximately 36% in both treatments, indicating effective removal. These results are consistent with those of a previous study that investigated the effect of washing, soaking, and malting processes on the level of phytic acid in sorghum [33]. This study reported reductions in phytic acid levels of 15.6%, 43.7%, and 54.9%, respectively. In addition, boiling maize products in distilled water and roasting them reduced the phytic acid levels by approximately 18.9% and 23.7%, respectively [34]. The reduction in phytic acid can be attributed to the heat treatment [35], which facilitates phytase hydrolysis [33,36]. A slight decrease in protein content was observed, with reductions of only 0.6% for APC-CA and 1.7% for APC-DW relative to UAPC. Protein content was not significantly affected by the processing method, as previously reported [37]. However, our results are in contrast to those of Sharma et al. [38], who reported a significant decrease in soybean protein content with the use of citric acid. This discrepancy may be due to differences in treatment duration. Both treatments significantly reduced lipid, ash, and fiber content. The reductions were about 35%, 9%, and 9%, respectively, for CA and 25%, 38%, and 19% for DW. The large reduction in lipids can be attributed to the increased activity of lipolytic enzymes. These enzymes facilitate the breakdown of triacylglycerols into simpler compounds, including fatty acids, sterol esters, and polar lipids [39]. Fiber content was reduced to a greater extent with a citric acid solution than with a distilled water solution. This finding is consistent with a study by Wanjekeche et al. [40], who reported a 26% and 52% reduction in dietary fiber content when *Mucuna pruriens* was boiled in citric acid and distilled water, respectively. In contrast, the results of [41], who investigated the effect of different concentrations of CA (1% or 3%) and a drying temperature of 65 °C on sweet potato flour, reported that the fiber content was significantly increased. In addition, the treatment process reduced the moisture content by 10% with citric acid and 20% with distilled water. These results are in agreement with those reported by Kumar et al. [28], who observed a 7% reduction in the initial moisture content of mushrooms after soaking them in a 5% citric acid solution.

#### 3.1.2. Mineral Content

With respect to mineral content, the treatments had contrasting effects: Iron content increased by 33.3% for APC-CA and 51.7% for APC-DW, while calcium content decreased by 19.8% and 32.7%, respectively. Zinc showed different trends, with a 47% decrease in APC-CA and a 47.6% increase in APC-DW. The phytate/iron and zinc molar ratios showed significant changes: the phytate/iron ratio was reduced by 51.6% for APC-CA and 58.8% for APC-DW, while the phytate/zinc ratio increased by 31% for APC-CA but decreased by 57.4% for APC-DW. However, our results, consistent with those of Alajaji and Adawy [42], who showed that boiling chickpeas in water results in a significant loss of calcium, with a reduction of up to 30%. Therefore, it is important to consider alternative cooking methods in order to preserve the calcium content. Another study on the effect of thermal processing on the mineral content of African walnut kernels showed a significant reduction of 71% in calcium content [43]. The bioavailability of iron is known to be influenced by various dietary components, including inhibitors such as phytic acid, tannins, dietary fiber, and calcium. Conversely, organic acids, on the other hand, are known to promote iron absorption [44,45]. Previous research suggests that the addition of phytase, citric acid, and ascorbic acid significantly improves iron availability [46]. Our results show that the pre-treatment methods positively affected the concentration of iron in the APC, which may have implications for its use in preventing nutritional deficiencies.

#### 3.1.3. Color Characteristics

Color is an important characteristic in the assessment of flour quality for the production of many products [47]. Color measurements revealed significant differences (*p* < 0.05) between treated and untreated samples. Lightness (L*) decreased slightly by about 2.5% for APC-CA and 0.8% for APC-DW. Redness (a*) showed a slight decrease of approximately 3.5% and 7% for APC-CA and APC-DW, respectively. However, there was no significant change in yellowing (b*) between treatments. Our results are similar to those of Ngoma et al. [48], who observed a decrease in L* value due to the application of citric acid. The decrease in L* may be due to various factors, such as alterations in carotenoid content, oxidative processes, caramelization reactions, or changes in phenolase activity [49]. The appearance of browning indicates a decrease in clarity, which is associated with the formation of brown pigments due to non-enzymatic reactions (Maillard reaction) generated by heat treatments [50]. The increase in b* (yellowness) when distilled water and citric acid are heated may be due to the Maillard reaction, which is a complex series of chemical reactions between amino acids (from proteins) and reducing sugars [51]. This reaction is responsible for the browning and development of a yellow to brown color in various foods when cooked or heated. The Maillard reaction results in the production of yellow and brown pigments, which contribute to an increase in color value. Overall, industrial food by-products represent an excellent source of functional ingredients [52].

#### 3.1.4. FTIR and Fluorescence Analysis

**Figure 1:**
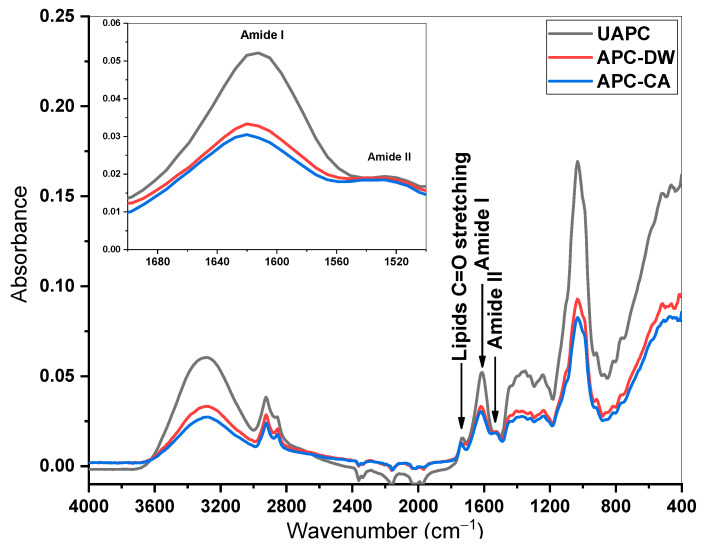
Normalized FTIR spectra of UAPC, APC-CA, APC-DW band derived from amide I.

**Figure 2:**
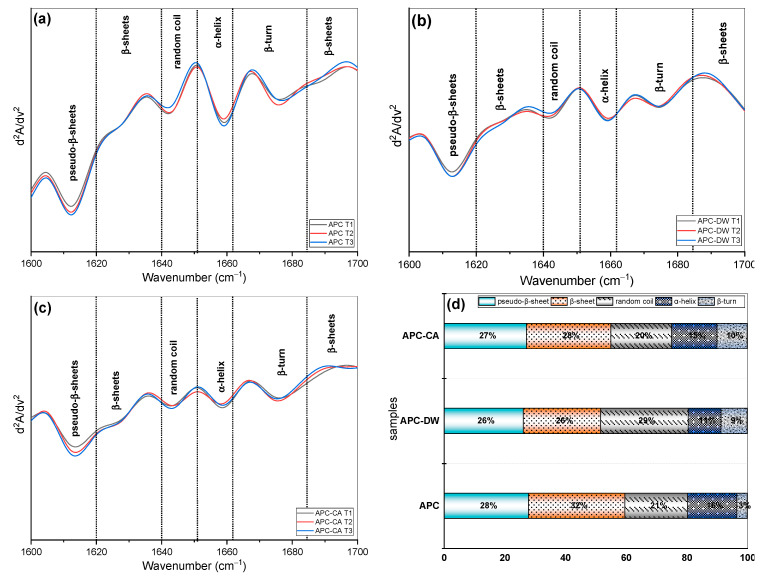
FTIR spectra, protein secondary structures (**a**–**c**), and relative percentages of each secondary structure (**d**).

In this work, for the first time, the secondary protein structure of APC was studied, revealing a profile similar to that of other oil cakes [53]. The APC protein is composed of approximately 41% albumins and 50% globulins of the total extractable protein. Disulfide bonds play a crucial role in stabilizing the protein structure, whereas gluten and gliadin are less crucial. Figure 1 shows the FTIR normalized full spectra of UAPC and treated APC. The peaks recorded in the region between 1300 and 1400 cm^−1^ are attributed to nitrogen, the main component of the proteins. The bands in the 1700–1500 cm^−1^ region are associated with protein molecules and are attributed to the amide I (80% CC = O stretch, 10% C–N stretch) and amide II (60%NH stretch, 30%C-N stretch, and 10% C–C stretch) bond vibrations [54,55]. The spectra were analyzed in the amide I region, which provides the most information about the secondary structure of proteins, and it was observed that heat treatment does not cause the transformation of major functional groups. The spectra of UAPC, APC-CA and APC-DW were similar. The boiling treatment of APC also affected the amide I and II absorbances, indicating changes in the protein structures due to the high temperature applied. Using the second derivative signal of the amide I (Figure 2), absorbances centered in the regions 1620–1644 and 1680–1700 cm^−1^ were attributed to β-sheet conformation, 1660–1680 cm^−1^ to β-turn conformation, 1650–1665 cm^−1^ to α-helix, 1644–1652 cm^−1^ to random coils [54,55], and ~1613 cm^−1^ to pseudo-β-sheet [56]. The relative proportions of each secondary structure for each sample are shown in Figure 2d. The protein in UAPC contained β-sheet (31.8% of the total secondary structure) as one of the major secondary structures. Heat treatments on distilled water led to a decrease in β-sheet, α-helix, while β-turn and random coil showed the opposite trend. Citric acid treatment led to a decrease in β-sheet and an increase in β-turn, while it did not significantly affect α-helix and random coil structure. Pseudo-β-sheet showed a small reduction for both treatments, from 28% to 26% and from 28% to 27% for APC-CA and APC-DW, respectively. Similar results were obtained in a study of the effect of heat treatment on the secondary structure of quinoa protein and camelina seed [57,58], probably due to the denaturation of the proteins due to the destruction of the hydrogen bonds between peptides leading to the transformation of the α-helix and β-sheet into β-turn and random coil [59,60]. In the case of citric acid treatment, which lowers the pH to an acidic range, a decrease in the content of β-sheet, α-helix, and pseudo-β-sheet content was observed, while the content of β-turn increased. However, these results contrast with those of Wang et al. [61], who reported that acidic conditions decreased α-helix content and increased β-sheet content. On the other hand, the study by Uranga et al. [62] observed that citric acid-incorporated fish gelatin/chitosan showed a decrease in the content of the β-sheet conformation and an increase in the α-helix/random coil. From this perspective, changes in the molecular conformation of the α-helix and β-sheet may be due to the high hydrogen bond content originating from citric acid. This citric acid can potentially replace the hydrogen bonds that typically form between different protein chains and between protein chains and water molecules. Consequently, the α-helix is increased, and other structural forms are decreased [63].

**Figure 3:**
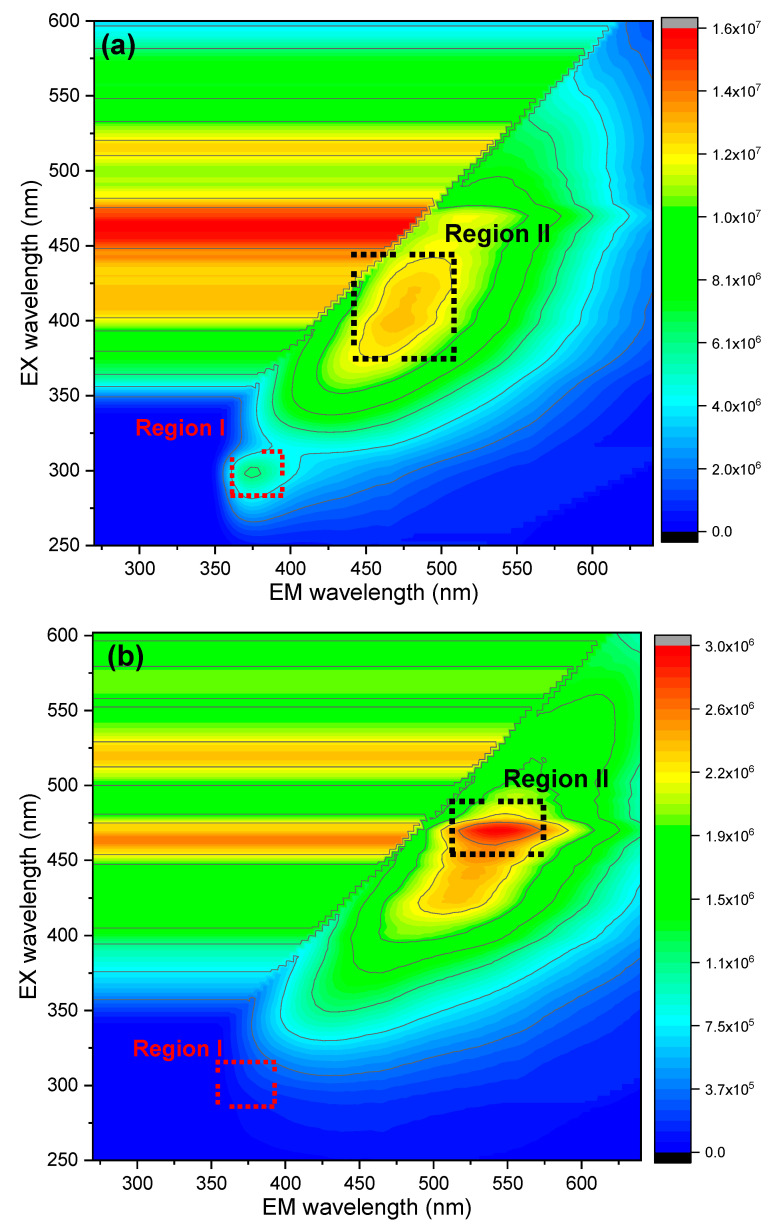

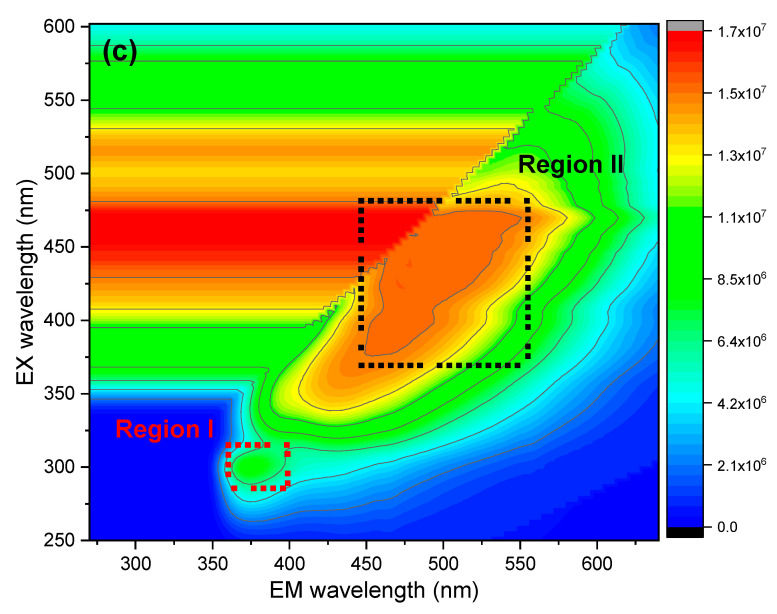
Fluorescence spectra corresponding to (**a**): UAPC, (**b**): APC-CA, and (**c**): APC-DW.

The study of intrinsic fluorescence provides a means of investigating chemical changes associated with the treatment process [64]. Figure 3 shows the fluorescence excitation and emission spectra of APC before and after treatment. Two characteristic regions are observed in the spectra of UAPC and APC-DW (regions I and II), while APC-CA is characterized by one region (region II). One region (region I), corresponding to tryptophan emission (386 nm < λ < 400 nm) [65], was found, while the other (region II), corresponding to fluorescence emission from non-protein compounds, with an Em peak located at (405 < λem < 650 nm), was contributed by riboflavin [66], with UAPC exhibiting region I (λEx = 280–310 nm, λEm = 360–390 nm) and region II (λEx = 370–440 nm, λEm = 440–510 nm). The treatment with distilled water caused a slight shift in tryptophan emission, resulting in region I (λEx = 285–315 nm, λEm = 360–400 nm) and region II (λEx = 370–480 nm, λEm = 445–555 nm) for APC-DW. In contrast, APC-CA exhibited only region II (λEx = 450–490 nm, λEm = 510–575 nm), which showed a shift compared to UAPC, while region I was absent. When the proteins unfold, the buried tryptophan residues are exposed to a more polar environment. Boiling with distilled water causes a shift in the maximum emission wavelength of tryptophan of about 10 nm. These emission spectrum results are in agreement with the findings of [67,68]. A shift in the fluorescence emission peak to longer wavelengths indicates that the tryptophan side chains move from a hydrophobic to a hydrophilic environment. In general, a maximum fluorescence emission wavelength (λ_max_) of <330 nm indicates that tryptophan residues are embedded in a hydrophobic environment, whereas λ_max_ > 330 nm indicates exposure to the aqueous environment [69]. The missing tryptophan fluorescence intensity region in APC-CA might be caused by more conformational changes at acidic pH, as described by Abdollahi and Pezeshk. The authors of [70,71] also reported that tryptophan fluorescence intensity in acid-produced protein isolate of kilka and rainbow due to the interaction of polysaccharides with the polar parts of proteins, which exposed more tryptophan residues and shifted them to a more hydrophobic environment under acidic conditions [72,73].

### 3.2. Characterization of Flour Blends

When comparing the proximate composition of different blends of whole wheat flour (WWF) with APC-CA and APC-DW, it was found that they were statistically different (Table 2). Protein, ash, lipids, fiber, and the essential minerals iron, zinc, and calcium were increased in all flour blends, indicating an improvement in nutritional value. Previous studies have also demonstrated an increase in nutritional quality with the incorporation of seeds and press cake flour by-products such as moringa seeds, flaxseed cake, hemp seeds, and beniseed [74,75,76,77]. However, a significant reduction in moisture content was observed. On the other hand, phytic acid levels and PA/Fe and PA/Zn molar ratios increased slightly, suggesting a potential reduction in mineral bioavailability despite higher mineral content. These trade-offs highlight the need for a balanced approach to optimize nutritional and functional qualities. The addition of APC-CA and APC-DW also significantly increased color values (L* and a*).

The mixograph provides invaluable insight into the dynamics of dough development and gluten formation. The duration of the mixing process is a crucial aspect in the baking industry, as it directly affects the quality of the final product.

Table 3 shows the variations in mixing peak height, mixing peak time, water absorption, and gluten strength for the WWF and the mixes with APC-CA and APC-DW. The results indicate that the WWF had the lowest water absorption of 63,40%. However, all the composite flours had higher water absorption as the proportion of APC content increased from 5% to 20%. This value further increased in both APC-CA and APC-DW with a range of 64.4% to 66.9% and 64.5% to 67.6%, respectively. Furthermore, it was observed that the use of distilled water for treatment resulted in an increase in water absorption of almost 1% compared to citric acid treatment for all composites. The mixing time required for dough development ranged between 3.06 and 4.72 min for different flour blends; it was determined that two treated argan press cakes have remarkably reduced mixing time. The lowest value of 3.90 min was registered for the flour blend containing 20% APC-CA, while the highest mixing time of 4.72 min was observed for WWF. It can be observed that the gluten straight of WWF decreased as the incorporation ratio increased.

The results of this study indicate that the enrichment of whole wheat flour with treated APC resulted in a decrease in mixing time and an increase in water absorption compared to whole wheat flour alone. This trend was also observed in bread made from wheat and peanut flour [78]. This can be attributed to the rapid formation of the gluten network in the dough, resulting in a reduced need for prolonged mixing due to the gluten-free flour. The observed increase in water absorption, which correlates with the higher APC-CA and APC-DW proportions, can be due to the increase in fiber content. This is because the fibers contain a greater number of hydroxyl groups, which form stronger bonds with water. The difference in water absorption between APC-DW and APC-CA may be explained by the citric acid effect, as previously mentioned by Gupta et al. [79]. This leads to changes in the rheological properties of the dough, a phenomenon that has been confirmed by previous research [80,81]. A shorter mixing time is often desirable as it reduces energy consumption and water absorption [27].

### 3.3. Characterization of Bread

**Figure 4:**
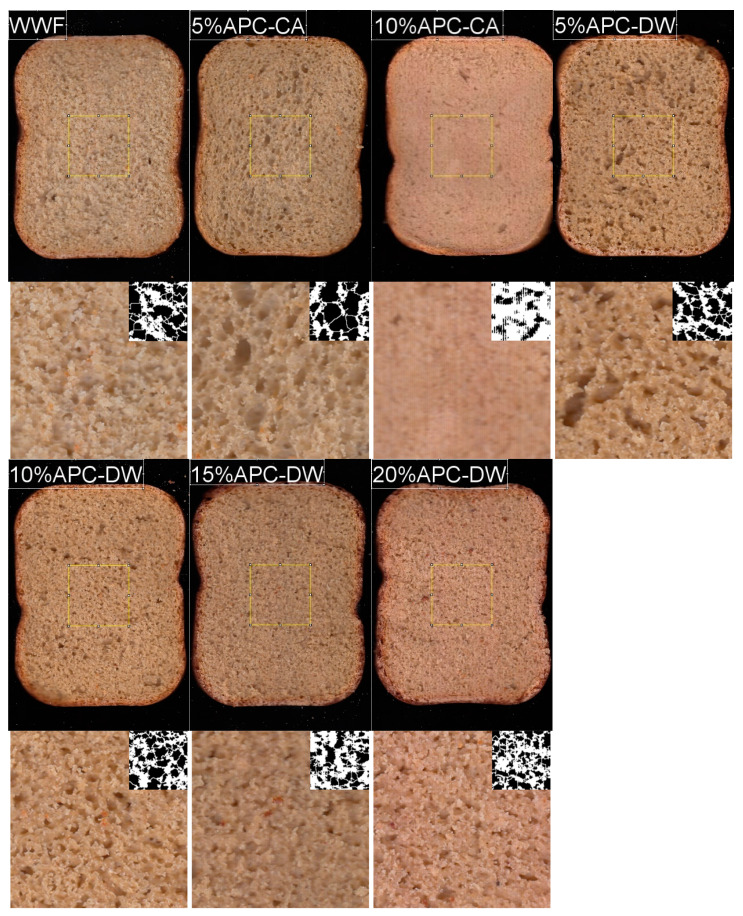
Aspect of bread obtained with WWF and addition of different quantities of APC-CA or APC-DW.

The bread obtained with WWF had the highest loaf volume with a value of 1150 cm^3^ (Table 4). The addition of 5% APC-CA did not affect the loaf volume, while the mixture of 10% APC-CA reduced the volume to 750 cm^3^. The loaf volume was reduced to 500 cm^3^ and 370 cm^3^ for the last two percentages, 15% and 20%, respectively, resulting in a deformed bread shape that prevented measurement of the other parameters. Blending of APC-DW caused a reduction in loaf volume from 850 to 650 cm³ as the rate increased from 5% to 20%. The color of bread the crumb changed from white to brown with a decrease in L* and b* values from 64.03 to 56.9 and from 25.38 to 21.44, respectively, while a* increased from 4.21 to 5.71 (Figure 4). Compared to the control bread, the blended breads containing 5% (APC-CA and APC-DW) showed a statistically significant decrease in the number of cells, while the average size increased. No significant changes (*p* < 0.05) were observed in the total area and porosity. Among the different breads tested (10% APC-CA, 10% APC-DW, 15% APC-DW, and 20% APC-DW), the bread with 10% APC-CA showed a significant reduction in porosity and total surface area, while a high number of cells and a reduced average cell size were observed.

The reduction in bread volume may be due to the high fiber content since fiber can also form hydrogen bonds with water and inhibit gluten network formation [82,83]. Another factor contributing to the reduction in bread volume is the high protein and lipid content, which results in a reduced swelling index [84,85,86]. Bread color is a critical factor in consumer acceptance [87], as it affects the overall perception of the product. Similar to our findings, the effects of different legume flour additions were cumulative on the original wheat flour color [88]. During baking, the thermal profile resulted in a decrease in the L* value of the composite bread crumb, while the crumb a* and b* values increased. This fact can be attributed to the reduction in moisture content, as observed by [89]. The incorporation of two treated APCs at a level of 5% (especially of APC-CA) resulted in a significant reduction in the number of cells in the bread and an increase in the average cell size, leading to an improvement in bread quality. However, when the incorporation level exceeded 10%, a deterioration in bread quality was observed. This suggests that the gluten matrix in the dough was unable to adequately accommodate gas molecules, resulting in the formation of dense, tightly packed cells during the fermentation and baking process [90].

## 4. Conclusions

The present study showed that a pre-treatment with hot water or a hot citric acid solution resulted in a significant reduction in saponin and phytic acid concentration in APC. The incorporation of both APC-CA and APC-DW resulted in an improved nutritional profile of the produced bread with an increased level of protein, lipids, fiber, and mineral content without significant changes in the rheological profile of the dough. Furthermore, the addition of 5% APC-CA can be incorporated into whole wheat bread without altering its quality. Finally, these results may contribute to the widespread use of APC in the food sector, capitalizing on its nutritional benefits while mitigating the undesirable aspects associated with its initial composition. Its use as an ingredient can serve as a cost-effective strategy for improving nutrition while offering an environmentally sustainable solution by minimizing food waste, bringing it into line with the principles of a circular economy.

## Figures and Tables

**Table 1:** Chemico-physical characteristics of untreated (UAPC) and treated (APC-CA, APC-DW) argan press cake.

Traits	UAPC	APC-CA	APC-DW
Saponin (mg/g)	4.71 ± 0.10 ^a^	0.80 ± 0.12 ^b^	1.20± 0.10 ^c^
Lipid (%)	20.30 ± 0.20 ^a^	15.30 ± 0.10 ^b^	13.20 ± 0.20 ^c^
Ash (%)	5.09 ± 0.02 ^a^	3.18 ± 0.05 ^b^	4.66 ± 0.03 ^c^
Fiber (%)	17.83 ± 0.20 ^a^	14.13 ± 0.10 ^b^	15.82 ± 0.20 ^c^
Protein (%)	47.70 ± 0.10 ^a^	47.40 ± 0.10 ^a^	46.88 ± 0.06 ^b^
Moisture (%)	9.00 ± 0.02 ^a^	7.33 ± 0.03 ^b^	8.01 ± 0.25 ^c^
Ca (mg/100 g)	690.5 ± 0.13 ^a^	553.4 ± 0.10 ^b^	464.7± 0.11 ^c^
Fe (mg/100 g)	8.7 ± 0.04 ^a^	11.6 ± 0.02 ^b^	13.2 ± 0.01 ^c^
Zn (mg/100 g)	6.3 ± 0.00 ^a^	3.1 ± 0.01 ^b^	9.3 ± 0.00 ^c^
Pythic acid (g/100 g)	1.63 ± 0.10 ^a^	1.05 ± 0.01 ^b^	1.03 ± 0.01 ^b^
Phytic Acid/Iron	15.98 ± 0.67 ^a^	7.73 ± 0.18 ^b^	6.58 ± 0.6 ^b^
Phytic Acid/Zinc	25.74 ± 0.06 ^a^	33.71 ± 0.87 ^b^	10.95 ± 0.06 ^c^
L*	80.21 ± 0.02 ^a^	78.18 ± 0.05 ^b^	79.53 ± 0.10 ^c^
a*	1.70 ± 0.01 ^a^	1.64 ± 0.01 ^b^	1.58 ± 0.01 ^c^
b*	12.21 ± 0.02 ^a^	12.28 ± 0.03 ^a^	12.24 ± 0.50 ^a^

Values followed by the different small letters are significantly different at *p* < 0.05 according to ANOVA and Tukey HSD test.

**Table 2:** Chemico-physical characteristics of mixture experiment flours.

Traits	WWF	5%APC-CA	10%APC-CA	15%APC-CA	20%APC-CA	5%APC-DW	10%APC-DW	15%APC-DW	20%APC-DW
Lipid (%)	1.71 ± 0.03 ^a^	2.69 ± 0.01 ^b^	3.37 ± 0.04 ^c^	3.92 ± 0.03 ^d^	4.89 ± 0.04 ^e^	2.17 ± 0.06 ^ab^	3.31 ± 0.03 ^c^	4.02 ± 0.06 ^d^	4.36 ± 0.05 ^d^
Ash (%)	1.54 ± 0.03 ^a^	1.55 ± 0.03 ^ab^	1.63 ± 0.02 ^ab^	1.72 ± 0.01 ^ac^	1.77 ± 0.01 ^c^	1.67 ± 0.01 ^b^	1.90 ± 0.01 ^d^	1.94 ± 0.01 ^de^	2.04 ± 0.02 ^e^
Fiber (%)	2.60 ± 0.10 ^a^	3.27± 0.05 ^b^	4.73 ± 0.03 ^c^	5.22 ± 0.05 ^de^	5.35 ± 0.06 ^ef^	3.38 ± 0.02 ^b^	4.90 ± 0.01 ^cd^	5.36 ± 0.04 ^ef^	5.69 ± 0.03 ^f^
Protein (%)	13.56 ± 0.03 ^a^	14.51 ± 0.02 ^b^	15.15 ± 0.01 ^c^	15.85 ± 0.01 ^de^	16.62 ± 0.01 ^ef^	14.17 ± 0.01 ^f^	15.13 ± 0.03 ^c^	15.62 ± 0.03 ^d^	16.33 ± 0.03 ^f^
Moisture (%)	11.40 ± 0.10 ^a^	11.25 ± 0.02 ^a^	10.92 ± 0.02 ^ab^	10.87 ± 0.01 ^ab^	10.64 ± 0.01 ^bc^	10.91 ± 0.01 ^ab^	10.55 ± 0.01 ^bc^	10.33 ± 0.01 ^cd^	10.11 ± 0.01 ^d^
Ca (mg/100 g)	15.15 ± 0.01 ^a^	15.24 ± 0.04 ^a^	16.48 ± 0.03 ^b^	17.28 ± 0.03 ^bd^	17.75 ± 0.09 ^bd^	15.92 ± 0.03 ^a^	17.28 ± 0.03 ^c^	18.71 ± 0.06 ^d^	19.88 ± 0.03 ^d^
Fe (mg/100 g)	3.92 ± 0.01 ^a^	4.51 ± 0.02 ^b^	5.42 ± 0.03 ^cd^	5.60 ± 0.02 ^cd^	5.65 ± 0.03 ^d^	5.01 ± 0.02 ^bc^	5.63 ± 0.01 ^d^	5.80 ± 0.01 ^d^	5.92 ± 0.01 ^d^
Zn (mg/100 g)	2.40 ± 0.02 ^a^	2.41 ± 0.01 ^a^	2.60 ± 0.03 ^a^	2.63 ± 0.02 ^a^	2.81 ± 0.03 ^ab^	3.42 ± 0.01 ^abc^	3.74 ± 0.02 ^abc^	3.93 ± 0.01 ^c^	4.12 ± 0.02 ^c^
Pythic acid (g/100 g)	0.62 ± 0.01 ^a^	0.73 ± 0.01 ^bc^	0.81 ± 0.02 ^cd^	0.88 ± 0.01 ^de^	0.95 ± 0.02 ^e^	0.69 ± 0.01 ^ab^	0.74 ± 0.02 ^bc^	0.83 ± 0.02 ^d^	0.92 ± 0.02 ^ef^
Phytic acid/iron	13.76 ± 0.20 ^ab^	13.69 ± 0.19 ^abc^	12.65 ± 0.31 ^bcd^	13.67 ± 0.16 ^ab^	14.39 ± 0.30 ^a^	11.86 ± 0.24 ^cd^	11.40 ± 0.31 ^d^	12.32 ± 0.31 ^bcd^	13.39 ± 0.29 ^ae^
Phytic acid/zinc	27.83 ± 0.42 ^a^	29.98 ± 0.51 ^a^	33.26 ± 0.71 ^b^	33.35 ± 0.38 ^b^	33.14 ± 0.70 ^b^	20.13 ± 0.30 ^d^	19.48 ± 0.52 ^d^	21.81 ± 0.52 ^cd^	22.88 ± 0.50 ^c^
L* (Flour)	77.14 ± 0.08 ^a^	85.31 ± 0.09 ^d^	85.27 ± 0.02 ^cd^	85.20 ± 0.57 ^cd^	84.29 ± 0.02 ^bc^	83.68 ± 0.08 ^b^	85.26 ± 0.11 ^cd^	84.60 ± 0.06 ^bcd^	84.17 ± 0.05 ^b^
a* (Flour)	1.79 ± 0.05 ^a^	0.69 ± 0.01 ^bd^	0.56 ± 0.01 ^c^	0.57 ± 0.02 ^c^	0.68 ± 0.01 ^bd^	0.69 ± 0.00 ^bd^	0.62 ± 0.01 ^bc^	0.73 ± 0.02 ^d^	0.76 ± 0.01 ^d^
b* (Flour)	12.56 ± 0.03 ^a^	12.78 ± 0.05 ^g^	12.10 ± 0.02 ^bd^	12.10 ± 0.01 ^bd^	11.97 ± 0.02 ^bc^	11.83 ± 0.03 ^c^	12.41 ± 0.07 ^aef^	12.65 ± 0.04 ^g^	12.46 ± 0.02 ^af^

Values followed by the different small letters are significantly different at *p* < 0.05 according to ANOVA and Tukey HSD test. WWF: whole wheat flour; APC-CA: treated Argan press cake with citric acid; APC-DW: treated Argan press cake with distilled water.

**Table 3:** Rheological parameters of whole wheat flour (WWF) and mixes with treated argan cake at different proportions (5, 10, 15, and 20%).

	WWF	5%APC-CA	10%APC-CA	15%APC-CA	20%APC-CA	5%APC-DW	10%APC-DW	15%APC-DW	20%APC-DW
Gluten straight (mL)	56.46 ± 0.1 ^a^	51.55 ± 0.2 ^b^	44.19 ± 0.1 ^c^	42.71 ± 0.5 ^cd^	39.28 ± 0.2 ^e^	47.62 ± 0.5 ^f^	41.73 ± 0.1 ^cde^	40.75 ± 0.5 ^de^	39.28 ± 0.0 ^c^
Mixing time (min)	4.72 ± 0.2 ^a^	3.98 ± 0.1 ^b^	2.96 ± 0.2 ^c^	3.96 ± 0.1 ^d^	4.40 ± 0.0 ^e^	4.12 ± 0.2 ^f^	3.08 ± 0.1 ^c^	3.06 ± 0.1 ^c^	3.16 ± 0.2 ^g^
Peak height (%)	43.8 ± 0.0 ^a^	47.6 ± 0.1 ^b^	50.0 ± 0.0 ^c^	47.6 ± 0.0 ^b^	50.7 ± 0.1 ^bc^	45.32 ± 0.0 ^e^	43.04 ± 0.0 ^a^	43.00 ± 0.0 ^a^	43.0 ± 0.0 ^a^
Water absorption (%)	63.4 ± 0.0 ^a^	64.4 ± 0.1 ^b^	65.0 ± 0.0 ^c^	66.0 ± 0.1 ^d^	66.9 ± 0.1 ^e^	64.5 ± 0.2 ^b^	65.4 ± 0.0 ^f^	66.4 ± 0.0 ^g^	67.6 ± 0.2 ^h^

Values followed by the different small letters are significantly different at *p* < 0.05 according to ANOVA and Tukey HSD test. WWF: whole wheat flour; APC-CA: treated argan press cake with citric acid; APC-DW: treated argan press cake with distilled water.

**Table 4:** Physical and textural characteristics of breads produced with the WWF and mixes with APC-CA and APC-DW.

	WWF	5%APC-CA	10%APC-CA	5%APC-DW	10%APC-DW	15%APC-DW	20%APCDW
Cells numbers	66.6 ± 0.03 ^a^	35.20 ± 0.0 ^b^	75.25 ± 0.0 ^c^	54.33 ± 0.02 ^d^	84.75 ± 0.03 ^e^	84.25 ± 0.03 ^e^	107.5 ± 0.03 ^f^
Total area	288.09 ± 0.02 ^a^	265.31 ± 0.01 ^a^	113.98 ± 0.01 ^b^	272.88 ± 0.02 ^a^	305.03 ± 0.02 ^c^	236.52 ± 0.0 ^d^	247.55 ± 0.02 ^e^
Average size (mm^2^)	4.4 ± 0.02 ^a^	7.83 ± 0.01 ^b^	1.53 ± 0.01 ^c^	5.01 ± 0.02 ^d^	3.59 ± 0.02 ^e^	2.81 ± 0.0 ^f^	2.32 ± 0.02 ^g^
Porosity (%)	32.01 ± 0.02 ^a^	29.47 ± 0.01 ^ab^	12.66 ± 0.01 ^c^	30.32 ± 0.02 ^a^	33.89 ± 0.02 ^a^	26.28 ± 0.0 ^b^	27.50 ± 0.02 ^b^
L* (Bread)	64.03 ± 0.02 ^a^	62.86 ± 0.01 ^b^	60.44 ± 0.02 ^c^	58.87 ± 0.02 ^d^	58.20 ± 0.02 ^d^	57.27 ± 0.03 ^d^	56.90 ± 0.03 ^d^
a* (Bread)	4.21 ± 0.02 ^a^	4.35 ± 0.01 ^a^	4.61 ± 0.02 ^b^	5.35 ± 0.03 ^c^	5.53 ± 0.03 ^d^	5.69 ± 0.02 ^e^	5.71 ± 0.02 ^e^
b* (Bread)	25.37 ± 0.03 ^a^	24.25 ± 0.03 ^b^	23.72 ± 0.01 ^c^	24.98 ± 0.01 ^d^	24.27 ± 0.02 ^b^	22.63 ± 0.02 ^e^	21.44 ± 0.02 ^f^
Loaf volume (cm^3^)	1150.00 ± 2.00 ^a^	1150.00 ± 1.00 ^a^	750.00 ± 2.00 ^b^	850.00 ± 1.00 ^c^	700.00 ± 1.00 ^d^	670.00 ± 1.00 ^e^	650.00 ± 2.00 ^f^

Values followed by the different small letters are significantly different at *p* < 0.05 according to ANOVA and Tukey HSD test. WWF: whole wheat flour; APC-CA: treated argan press cake with citric acid; APC-DW: treated argan press cake with distilled water.

## Data Availability

The original contributions presented in this study are included in the article. Further inquiries can be directed to the corresponding author.
